# Polyphasic Characterization of *Acetobacter indonesiensis* UNPADCC 01‐5 Isolated From a Traditional Fermented Food, Oncom Merah

**DOI:** 10.1111/1758-2229.70346

**Published:** 2026-06-04

**Authors:** Yolani Syaputri, Erisa Zahra, Rianti Nur Azzahra, Annisa, Diffa Amanda Iswhara, Felice Olivia Lengkey, Syiffa Fauzia, Xiwu Jia, Asri Peni Wulandari, Ratu Safitri

**Affiliations:** ^1^ Department of Biology, Faculty of Mathematics and Natural Sciences Universitas Padjadjaran Jatinangor West Java Indonesia; ^2^ Center for Bioprospection of Natural Fibers and Biological Resources, Faculty of Mathematics and Natural Sciences Universitas Padjadjaran Bandung West Java Indonesia; ^3^ Center of Exploration and Utilization of Natural Resources and the Environment Universitas Padjadjaran Jatinangor West Java Indonesia; ^4^ Research Center for Chemistry, National Research and Innovation Agency of Indonesia Jakarta Indonesia; ^5^ Department of Food Science and Engineering Wuhan Polytechnic University Wuhan China

**Keywords:** acetic acid bacteria, *Acetobacter indonesiensis*, fermented foods, oncom merah, probiotic potential

## Abstract

*Acetobacter indonesiensis* UNPADCC 01‐5 is a recently discovered acetic acid bacterium isolated from fermented food, oncom merah. With its current lack of polyphasic approach in classification, this study aims to provide the first morphological, physiological, biochemical and molecular analyses. These analyses are achieved by morphological and physiological characterization, Kirby–Bauer antibiotic susceptibility, biochemical profiling, gas chromatography–mass spectrometry and whole‐genome sequencing (WGS). The results found that *A. indonesiensis* UNPADCC 01‐5, a non‐motile cell, is a gram‐negative, short rod‐shaped cell that formed cream‐white colonies with 0.3–1.3 in diameter. It exhibited environmental resilience, including acid‐tolerant, halotolerant and thermotolerant. Against antibiotics, it showed resistance towards chloramphenicol. Biochemical tests revealed its ability to ferment glucose, rhamnose, melibiose and arabinose, as well as to produce bioactive metabolites such as acetoin and acetic acid. WGS identified potential gene expressions associated with acetic acid production, stress tolerance, nitrogen fixation, hydrocarbon degradation and heavy‐metal resistance. WGS analysis revealed no detectable genes associated with human virulence factors or pathogenic secretion systems. The results suggested that *A. indonesiensis* UNPADCC 01‐5 from oncom merah possessed adaptive traits which, with future assessments and industrial‐scale tests, hold potential as a functional/adjunct culture for acidic fermentations and a biofunctional agent.

## Graphical abstract

The polyphasic approach (morphology, physiology, biochemistry and genetics) was used to characterize *Acetobacter indonesiensis* strain UNPADCC 01‐5, which was isolated from oncom merah. The strain's potential as a culture for acidic fermentations and a biofunctional agent was supported by its adaptive characteristics, which included acid tolerance, metabolite production and functional genes for stress resistance.

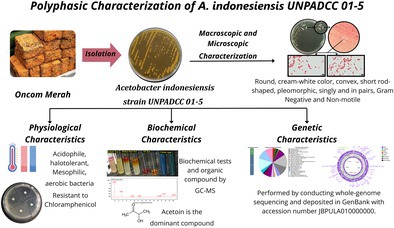

## 1 Introduction

Oncom merah is a traditional Indonesian fermented food made from tofu processing waste, cassava pulp and peanut press cake (Aini et al. 2020; Wikanta 2019). The fermentation process relies on the natural microbiota derived from its raw ingredients, along with the inclusion of a starter culture, particularly *Neurospora sitophila* (Wikanta 2019). These raw ingredients contain a high amount of protein that makes it a nutritious substrate. Furthermore, its natural method of fermentation with less sanitary conditions supports growth of various microorganisms in the environment (Wijaya et al. 2024). In addition, microorganisms within this unique microbial community form a complex network of interactions, resulting in mutualistic, parasitic, or amensalistic relationships. Acetic acid bacteria (AAB), including *Acetobacter* species, are known to play important roles in this microbial network through their ability to produce acetic acid, which can inhibit competing microorganisms while potentially supporting acid‐tolerant species (Lynch et al. 2019). The acidic environment created by *Acetobacter* may provide selective pressure that shapes the overall microbial community composition during oncom fermentation, influencing both the fermentation process and final product characteristics (Han et al. 2024). Previous studies have reported the presence of various bacteria in oncom merah, including lactic acid bacteria (Kurniati et al. 2021), and several *Bacillus* species (Syahbanu 2023; Wijaya et al. 2024). A recent study isolated *Acetobacter indonesiensis* UNPADCC 01‐5 from oncom merah and analysed its potential role in this complex fermented food ecosystem.

Several preliminary studies have reported the isolation of *A. indonesiensis* from various fermented foods globally, including Thai fermented rice (Pitiwittayakul et al. 2015; Tanasupawat et al. 2011), kombucha (Rodriguez Rey et al. 2024) and traditional Iranian dairy products (Haghshenas et al. 2015). As a member of the AAB, certain strains of *A. indonesiensis* and related species have demonstrated probiotic‐associated traits, such as tolerance to low pH and bile salts (Haghshenas et al. 2015; Wang et al. 2024). Furthermore, certain AAB strains had been proven to exhibit a wide range of beneficial properties, including in vitro anticancer, anti‐inflammatory and antimicrobial activities (Fabricio et al. 2023; Hata et al. 2023). Selected AAB strains isolated from fermented foods have been reported to enhance or stabilize antioxidant activity, produce various health‐promoting organic acids and synthesize valuable compounds such as miglitol (an antidiabetic agent), vitamin C and exopolysaccharides, including levan (a prebiotic) and bacterial cellulose (Fabricio et al. 2023; Hata et al. 2023; Morales et al. 2023; Neffe‐Skocińska et al. 2023; Wang et al. 2024).

*A. indonesiensis* UNPADCC 01‐5 isolated from oncom merah was investigated in this study. While *A. indonesiensis* has been reported in other fermented foods, this is the first report of its isolation from oncom merah and the first comprehensive polyphasic characterization of the species from this Indonesian fermented food matrix, providing insight into strain‐specific adaptations shaped by the fermentation environment. Each strain of bacteria from different sources had diverse characteristics and metabolic abilities (Morgan et al. 2021). Moreover, characteristic traits including morphological, physiological and biochemical features are expressed by bacterial strains. Genotypic traits responsible for differentiating strains based on unique genetic content played a crucial role in comprehensive characterization (Liu et al. 2020).

Sole reliance on conventional molecular identification, such as 16S rRNA gene sequencing, is often insufficient for distinguishing closely related species and does not provide insight into the functional attributes of an isolate. Conversely, phenotypic characterization alone can be ambiguous due to the influence of environmental factors on gene expression (Xiang et al. 2025). To overcome the limitations inherent in these single‐method approaches, a polyphasic characterization is essential. By integrating morphological, physiological and biochemical features with genomic analysis, this strategy provides a consensus classification that is more robust and accurate, effectively bridging the gap between genetic potential and phenotypic expression (Arif and Agusta 2015). Complementing these methods, genomic and metabolomic analyses are powerful molecular tools used to detect specific genes and proteins as well as identify bioactive compounds within bacterial isolates. The tools further provided essential biochemical information, which helped monitor gene function (Roring 2019; Viersanova and Purwanto 2021).

While accurate strain identification is fundamental to food safety and environmental monitoring, exploring indigenous fermented foods like oncom merah offers a broader opportunity to expand the global understanding of food microbiome diversity. These unique ecological niches often harbour wild‐type strains with distinct metabolic and adaptive traits that differ significantly from industrialized cultures (Sooresh et al. 2023). Characterizing *A. indonesiensis* from this specific matrix contributes to microbiome science by revealing how traditional fermentation environments shape bacterial stress tolerance and metabolic versatility. Furthermore, identifying robust, stress‐tolerant strains from non‐dairy sources is essential for functional food innovation, offering novel candidates for starter cultures and potential probiotics that meet the growing global demand for resilient and diverse microbial resources. Therefore, this study aimed to characterize *A. indonesiensis* UNPADCC 01‐5 isolated from oncom merah using a polyphasic approach. We hypothesize that adaptation to the acidic, nutrient‐rich oncom merah environment confers strain‐specific metabolic and stress tolerance traits with potential relevance for fermented food and industrial biotechnological applications.

## 2 Materials and Methods

### 2.1 Materials

The materials used were Acetobacter Agar (AA), nutrient Agar (NA), iodine solution, nutrient broth (NB), NaCl, NaOH, HCl, Mueller Hinton agar (MHA), Moeller decarboxylase broth, arginine, L‐lysine, ornithine, Simmon citrate agar slants, triple sugar‐iron agar slants, urea broth, methyl red‐Voges–Proskauer (MR‐VP) broth, alpha‐naphthol, KOH, nutrient gelatine, glucose, mannitol, inositol, sorbitol, rhamnose, sucrose, melibiose, amygdalin, arabinose broth and ortho‐nitrophenyl‐β‐D‐galactopyranoside (ONPG) (Himedia, Maharashtra, India). Furthermore, this study used crystal violet solution, Oxidase Test Strip, (Merck, Darmstadt Germany), 95% ethanol (Fulltime, Anqing, China), safranin solution (Indoreagen, Jakarta, Indonesia), 3% hydrogen peroxide (H_2_O_2_) (Onemed, East Java, Indonesia), amoxicillin, erythromycin, tetracycline and chloramphenicol (Thermo Fisher Scientific, Massachusetts, USA), Wizard(R) Genomic DNA Purification Kit (Promega, Madison, USA).

### 2.2 Macroscopic and Microscopic Characterization

The isolate was purified by inoculation onto AA medium and incubated for 24 h at 30°C under aerobic conditions (Pulungan and Tumangger 2018). In addition, macroscopic colony morphology was observed detailing colour, size, shape and margin to confirm the expected characteristic features of the bacteria. The microscopic characteristics of the observed cell comprised shape, structure, size and gram staining (Cappuccino and Welsh 2018; Paray et al. 2023). Gram staining was performed following standard procedure using standard reagents and observed under 1000× magnification (Leica DM500 with Flexacam i5) (Dwimartina et al. 2021). All these morphological characteristics were conducted in more than 10 replications.

### 2.3 Motility Test

The motility testing was performed by using a semi‐solid NA medium (0.4% w/v) in test tubes under aerobic condition. Additionally, *A. indonesiensis* UNPADCC 01‐5 was inoculated by a single vertical stab in the centre of the semi‐solid NA medium using an inoculation needle and then incubated for 24 h at 37°C (Simanjuntak and Naibaho 2023). Motility test was conducted in triplicate.

### 2.4 Physiological Characteristics

*A. indonesiensis* UNPADCC 01‐5 was cultured in NB medium and incubated aerobically for 24 h under varying conditions of pH (3.0, 4.5, 6.5 and 9.0), NaCl concentration (4%, 6% and 8%) and incubation temperature (±27°C–30°C, 37°C and 45°C). The pH was adjusted by adding either 32% HCl or NaOH, with the final value confirmed using a pH meter. Meanwhile, during the salinity tolerance testing, NaCl was added to the medium at the specified concentrations. Bacterial growth was monitored 24 h after incubation by measuring the optical density (OD) at 600 nm using Spectrophotometer X Series Vis 50 DA‐X. Preliminary research reported that uninoculated medium was adopted as a control for each treatment (Arivo and Annissatussholeh 2017; Sadikin et al. 2021). All experiments were conducted in triplicate.

### 2.5 Antibiotic Sensitivity Test

Antibiotic susceptibility testing was carried out to assess the sensitivity of *A. indonesiensis* UNPADCC 01‐5 to specific antibiotics using the Kirby‐Bauer disk diffusion method, following the criteria put forward by the Clinical and Laboratory Standards Institute (CLSI). The following antibiotics: amoxicillin (30 μg), tetracycline (30 μg), chloramphenicol (30 μg) and erythromycin (15 μg) were tested. During the experiment, MHA medium served as the growth medium. The bacterial culture was suspended in 0.9% NaCl to achieve a turbidity equivalent to the 0.5 McFarland standard. However, this suspension was inoculated evenly on the surface of the MHA plate using a sterile cotton swab. Antibiotic discs were placed on the medium surface, and the plates were incubated at 37°C for 24 h. A vernier caliper was used to measure the inhibition zones around the discs (Khusuma et al. 2019). Antibiotic resistance test was conducted in triplicate.

### 2.6 Catalase Test

The catalase test was performed to determine the enzymatic activity of the bacterial cells using a 3% H_2_O_2_ solution (Onemed, East Jawa, Indonesia). Based on this perspective, a loopful of 24‐h bacterial culture from NB was placed on a glass slide. One to two drops of 3% H_2_O_2_ were added to the culture and observed for a few moments. The formation of bubbles showed a positive result (catalase‐positive), and its absence implied a negative outcome (Simanjuntak and Naibaho 2023). Catalase assay was conducted in triplicate.

### 2.7 Oxidase Test

The oxidase test was conducted by smearing a single bacterial colony on an Oxidase Test Strip (Merck, Darmstadt, Germany) using an inoculation loop. Colour changes were observed immediately after the smearing process. A positive result was shown by the development of a deep purple‐blue colour on the smeared area, while no colour change (remaining white) was considered a negative outcome (Cappuccino and Welsh 2018). Oxidase assay was conducted in triplicate.

### 2.8 Biochemical Characteristics

Bacterial cultures were grown in NB for 24 h, and the inoculum was standardized to 0.5 McFarland turbidity. All biochemical assays were incubated at 37°C for 24 h before interpretation of results. Biochemical Test Various were performed based on the methods outlined in Microbiology, A Laboratory Manual Eleventh Edition. These assays utilized Moeller's decarboxylase broth, amino acids (arginine, L‐lysine and ornithine), Simmon's citrate, triple sugar‐iron agar slants, urea broth, MR‐VP broth, α‐naphthol, KOH, nutrient gelatin medium and various carbohydrates including glucose, mannitol, inositol, sorbitol, rhamnose, sucrose, melibiose, amygdalin, arabinose and ONPG (Cappuccino and Welsh 2018). Each experiment was conducted in duplicate.

### 2.9 Analysis of Organic Compounds

Organic compounds in the bacterial sample were identified using gas chromatography–mass spectrometry (GC–MS) (Saravanakumar et al. 2021). Furthermore, the *A. indonesiensis* UNPADCC 01‐5 was cultured in 50 mL of NB, incubated for 5 days, and mixed at 150 rpm. The culture was centrifuged at 4000 rpm for 15 min, followed by the separation of the pellet from the supernatant (Octarya et al. 2022). The analysis of the organic compounds was conducted at the Central Laboratory of Universitas Padjadjaran using a GC–MS Agilent 7890A (Agilent Technologies, Santa Clara, USA). Organic acid analysis was conducted in duplicate.

### 2.10 Genetic Characteristics

Genomic DNA of *A. indonesiensis* UNPADCC 01‐5 was extracted using the Wizard Genomic DNA Purification Kit according to the manufacturer's protocol. Whole‐genome sequencing (WGS) was performed on the MGI DNBSEQ‐G400 platform (MGI Tech, Guangdong, China), generating paired‐end reads in FASTQ format. The quality of the raw sequence reads was assessed using FastQC v0.12.1. Adapter sequences and low‐quality bases (Phred score < Q30) were trimmed using Trimmomatic 0.40. Clean reads were assembled de novo using SPAdes genome assembler v4.2.0 with k‐mer sizes of 21, 33, 55 and 77. Contigs shorter than 200 bp and with coverage below 30× were removed from the final assembly. Genome completeness was evaluated using BUSCO 6.0.0 against the rhodospirillales_odb10 lineage dataset. Genome contamination and completeness were further assessed using CheckM lineage_wf (Galaxy Version 1.2.4+galaxy2) on the Galaxy platform. Assembly statistics, including genome size, GC content, number of contigs and N50 value, were calculated to evaluate assembly quality. Genome annotation was performed using Prokka v1.14.6 with default parameters. Functional annotation was further refined using the RASTtk pipeline via the RAST server (https://rast.nmpdr.org). Metabolic pathway reconstruction was conducted using KEGG mapper version 3.1 BlastKOALA against the KEGG database. Genome visualization was performed using the CGView Server (https://proksee.ca/).

The 16S rRNA gene sequence was extracted from the assembled genome and compared against the EzBioCloud (https://www.ezbiocloud.net/) database using BLAST searches. A similarity threshold of ≥ 99% was used for preliminary species‐level identification. Reference 16S rRNA gene sequences of closely related type strains were retrieved from NCBI (http://www.ncbi.nlm.nih.gov/). Multiple sequence alignment was performed using ClustalW, and a maximum‐likelihood phylogenetic tree was constructed in MEGA with 1000 bootstrap replicates.

Whole‐genome sequences of closely related *Acetobacter* species were downloaded from the NCBI database. Core genes were identified using Roary (Galaxy version 3.13.0+galaxy3). The core gene alignment was used to construct a phylogenomic tree using the IQ‐TREE web server (http://iqtree.cibiv.univie.ac.at) and the resulting tree was visualized in MEGA12. Average Nucleotide Identity (ANI) was calculated using FastANI 1.34. Digital DNA–DNA hybridization (dDDH) values were estimated using the Genome‐to‐Genome Distance Calculator (https://ggdc.dsmz.de/ggdc.php). Species delineation thresholds of ≥ 95%–96% for ANI and ≥ 70% for dDDH were used to evaluate genome‐relatedness.

### 2.11 Data Analysis

Quantitative data presented as the mean ± standard error (*n* = 3) were analysed using the one‐way analysis of variance (ANOVA) at a 95% confidence level (*α* = 0.05). In addition, a post hoc Tukey test was carried out to identify significant differences (*p* ≤ 0.05), and all statistical analyses were conducted using GraphPad Prism software version 10.

## 3 Results and Discussion

### 3.1 Morphological Characteristics

This analysis included macroscopic and microscopic observations, as well as a motility test. Moreover, the results of the morphological characterization for *A. indonesiensis* UNPADCC 01‐5 are shown in Table 1.

**TABLE 1:** Morphological characteristics of *A. indonesiensis* UNPADCC 01‐5 compared to previous descriptions in the literature.

Characteristics	UNPADCC 01‐5	Reference description
Morphological colony	Round, cream‐white colour, convex colonies with entire margins 0.3–1.3 mm in diameter	Round, convex or raised elevations, possessing pale colours such as white, yellow, or brown (Ester 2022).
Gram staining	Gram negative	Gram‐negative (Thanh Binh et al. 2017)
Size	0.5–0.8 × 1–1.5 μm	0.8–1.0 × 1.8–2.0 μm (Lisdiyanti et al. 2000)
Shape	Short rod‐shaped, pleomorphic	Elliptical, pleomorphic (Thanh Binh et al. 2017; Kohlmann et al. 2016)
Cell configuration	Singly and in pairs	Singly and pairs or clusters (Lisdiyanti et al. 2000; Thanh Binh et al. 2017)
Motility	Non‐motile	Motile (Ndoye et al. 2007)

Morphological analysis of *A. indonesiensis* UNPADCC 01‐5 revealed several characteristics that distinguish it from reference isolates. Colonies grown on AA medium measured 0.3–1.3 mm in diameter after 24 h of incubation at 30°C, which is notably smaller than the 2–3 mm colony size previously reported for *A. indonesiensis* strains from other sources. Microscopic observations showed cell dimensions ranging from 0.5–0.8 × 1.0–1.5 μm. These dimensions are smaller than those of the type strain (0.8–1.0 × 1.8–2.0 μm) (Lisdiyanti et al. 2000), with cells predominantly occurring singly or in pairs, as documented in Data S1. Furthermore, microscopic examination indicated a pleomorphic nature, consistent with reports by Kohlmann et al. (2016) that characterize the majority of these cells as Gram‐negative rods.

Gram staining confirmed the Gram‐negative characteristics typical of AAB. Colonies appeared cream‐white, convex and possessed entire margins. While these features are consistent with general Acetobacter morphology, slight differences in colony opacity were observed compared to previously described strains (Ester 2022).

Motility assessment using the semi‐solid agar stab method showed growth confined strictly to the inoculation line, indicating a non‐motile phenotype. Although confirmatory analyses such as flagellar staining or electron microscopy were not performed, genomic analysis revealed an absent flagellar biosynthesis gene cluster (flagellin gene; *flaA, fliC* and *flhA*), which is consistent with the observed phenotypic result. This non‐motile characteristic represents a variation within the species, as members of the genus *Acetobacter* can be either motile with peritrichous flagella or non‐motile (Ester 2022).

### 3.2 Physiological Characteristics

A critical aspect of bacterial analysis is physiological characterization, essential for predicting a bacteria's ecological distribution and function. It provided insight into phenotypic adaptations to environmental stress, including the impact on metabolic behaviour in intracellular surroundings (Cayron and Lesterlin 2019; Celeste et al. 2022; York 2017). These physiological tests were conducted to evaluate the ecological adaptability and environmental stress tolerance of *A. indonesiensis* UNPADCC 01‐5. The assessment focused on the strain's capability to withstand varying temperatures, pH levels and NaCl concentrations. Growth under extreme pH and elevated salinity represents fundamental stress tolerance traits that are relevant to both the strain's ecological adaptation in fermented food environments and its potential for biotechnological applications (Khushboo et al. 2023), as shown in Table 2.

**TABLE 2:** Physiological characteristics of *A. indonesiensis* UNPADCC 01‐5.

Characteristics	Results
pH tolerance	Acid‐tolerant
Salinity tolerance	Non‐halophilic, halotolerant (low to moderate tolerance)
Temperature tolerance	Mesophilic, thermotolerant
Catalase	Positive, aerobic bacteria
Oxidase	Negative

Catalase activity, indicated by a positive test result, confirms the strain's aerobic metabolism and its capacity to neutralize hydrogen peroxide (H_2_O_2_), a toxic byproduct of aerobic respiration (Murugan et al. 2026). This enzymatic function is ecologically significant as it enables survival in oxygen‐rich environments and protects against oxidative stress during fermentation processes (Nandi et al. 2019). The negative oxidase test result, characteristic of the genus *Acetobacter*, indicates the absence of cytochrome c oxidase in the terminal electron transport pathway (Adamopoulou et al. 2026; Shimada et al. 2026). Together, these enzymatic profiles are consistent with AAB metabolism and provide fundamental biochemical markers for strain characterization, though their direct relevance to probiotic or specific ecological functions requires further investigation in the context of host–microbe or microbe–microbe interactions.

#### 3.2.1 Optimal Growth Condition

**FIGURE 1:**
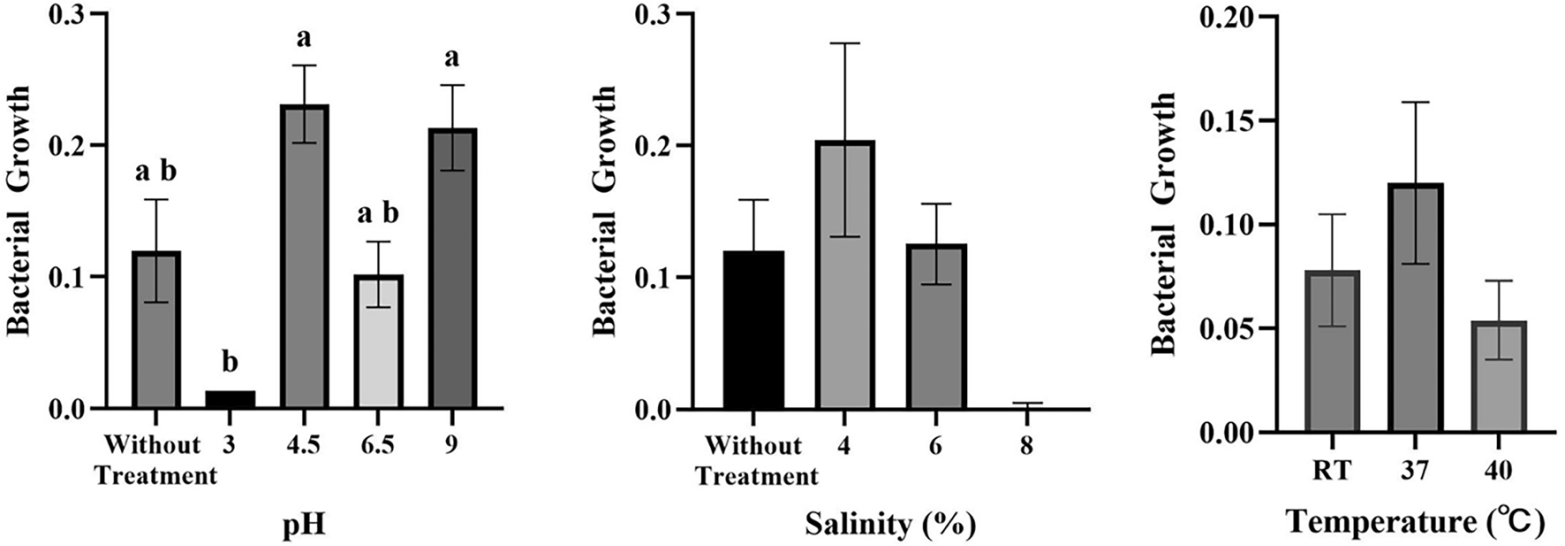
Bacterial growth (measured at OD600) of *A. indonesiensis* UNPADCC 01‐5 under varying pH, salinity and temperature conditions after 24 h. Data are presented as the mean ± standard error (*n* = 3). RT means room temperature (±27°C–30°C).

Optimal growth conditions are the best or most suitable environmental conditions that allow bacteria to grow and reproduce at maximum rate. In this research, the growth of *A. indonesiensis* UNPADCC 01‐5was evaluated to determine the optimal pH, salinity and temperature. These parameters are critical factors influencing bacterial survival and biomass accumulation. The bacteria were inoculated into NB medium adjusted to various pH values of 3, 4.5, 6.5 and 9. Growth optimization under varying salinity was performed by supplementing NB medium with different salt concentrations (4%, 6% and 8%). The bacteria were inoculated into NB medium and incubated at 37°C and 45°C, including room temperature (±27°C–30°C) for 24 h. Optimal temperature reflects the natural environmental conditions of the microorganism's habitat (Riskawati 2016). Figure 1 shows the growth of *A. indonesiensis* UNPADCC 01‐5 at various pH, salinity and temperature.

Optimal growth of *A. indonesiensis* UNPADCC 01‐5 after 24 h was denoted by the highest absorbance in the medium with an initial pH of 4.5, classified as an acid‐tolerant bacterium. However, the medium with an initial pH of 9 experienced a decrease during fermentation, reaching a final pH of 6.5 after the 24‐h incubation period. The growth observed at pH 3 showed a significant difference compared to pH 4.5 and 9, while the growth at pH 6.5 and without treatment (pH 6.8–7.2) did not exhibit a significant difference from the other groups. *A. indonesiensis* UNPADCC 01‐5 is not a halophilic bacterium, although it showed tolerance to certain salt concentrations, particularly 4% and 6%. This led to the classification as a halotolerant bacterium with low to moderate tolerance range. Growth tests carried out at varying salinities showed that *A. indonesiensis* was able to grow at NaCl concentrations of 4 to 6%, with optimal growth observed at the lowest concentration (4%). However, at a concentration of 8%, the bacteria were unable to grow, as shown by a significant decrease in optical density. The isolate was able to grow across all tested temperatures, ranging from 27°C to 45°C. The highest growth activity within 24 h was successively recorded at 37°C, followed by room temperature (27°C–30°C), with the lowest growth at 45°C. The One‐Way ANOVA analysis showed no significant difference in growth (OD600) among the salinity and temperature treatments (*p* > 0.05).

Bacteria must maintain a stable cytoplasmic pH to preserve the structural and functional integrity of its proteins, essential for cell growth. Some bacteria can maintain a neutral intracellular pH despite significant decreases in environmental pH. However, other species that lacked effective H^+^ extrusion or ion absorption systems failed to maintain homeostasis (Zhou et al. 2024). A disrupted proton motive force led to ion leakage, including protons, which lowered the cytoplasmic pH under acidic conditions. The inability to restore physiological pH triggered structural damage to the cell, eventually causing the disintegration of the cytoplasmic membrane (Porto‐Figueira et al. 2022).

In this study, the optimal growth of *A. indonesiensis* UNPADCC 01‐5 at pH 4.5, contrasted with significantly reduced growth at pH 3 (Figure 1), serves as a direct phenotypic indicator of its acid adaptability. The higher absorbance at pH 4.5 suggests these moderately acidic conditions are most favourable for its cellular activity, whereas the extreme acidity of pH 3 imposes clear physiological constraints. Furthermore, the strain's ability to grow across a broad initial pH range (3–9) and its observation of decreasing the medium pH from 9 to approximately 6.5 during the 24‐h incubation period reflects active metabolism and organic acid production. This behaviour is consistent with the established metabolic traits of the genus *Acetobacter*, which acidifies its environment during growth (Pitchai and Ramasamy 2026). These results indicate that while the strain is acid‐tolerant and physiologically adapted to its ecological niche in oncom merah, its growth performance is optimized under moderately acidic conditions.

Arifuzzaman et al. (2014), reported that the genus *Acetobacter* can grow at a pH of 4.5 to 7, with optimum growth observed between 5.0 and 6.5. During fermentation, *Acetobacter* produces an acidic environment through the oxidation of methanol to acetic acid, which acts as an inhibitory agent against other non‐acidophilic microorganisms (Permana et al. 2021). Meanwhile, tolerance to an acidic environment is a crucial characteristic for probiotic bacteria to grow, multiply and survive in the digestive tract. Probiotics must be able to withstand acidic conditions and bile salts to colonize the host gut. Most external microorganisms die on entering the digestive system due to exposure to stomach acid. Therefore, the ability to survive at low pH (1.0–3.0) and high bile salt concentrations (0.3% w/v) for relatively 90 min is a major requirement for probiotic candidates (Khushboo et al. 2023).

As an acid‐tolerant microorganism, *A. indonesiensis* UNPADCC 01‐5 has potential applications in the production of safe and high‐quality fermented foods. Microorganisms that could withstand extreme pH conditions were also proven to be suitable for industrial applications. Many acidophilic microorganisms, especially the thermophilic, naturally produced enzymes capable of degrading polymeric and oligomeric carbon sources. This led to the superior applications in lignocellulosic biorefineries, including food and textile industries (Icer et al. 2023). While *Acetobacter* sp. have been reported to contribute to industrial processes such as biorefineries and textile applications through the production of oxidative and carbohydrate‐active enzymes, specific enzymatic assays were not performed in this study (Kiefer et al. 2021; Provin et al. 2021). Consequently, the potential involvement of *A. indonesiensis* UNPADCC 01‐5 in such industrial applications remains to be confirmed. The relevance of this strain to industrial biotechnology should be regarded as a subject for dedicated functional and enzymatic validation in future studies.

In this study, NaCl was supplemented into the growth medium as a standardized stressor to evaluate the general osmoadaptive response of the bacterial strain. High salinity imposes significant osmotic pressure, which can induce cell damage, increase the production of reactive oxygen species (ROS) and inhibit essential metabolic processes (Bisson et al. 2023). Therefore, the ability to tolerate NaCl serves as an indicator of the strain's physiological robustness. This adaptive trait is particularly relevant for survival during food fermentation processes characterized by high osmotic pressure and reflects the organism's general resilience to environmental stress (Sionek et al. 2024).

Further, extremely high temperatures caused irreversible protein denaturation, although they inhibited enzyme activity when too low. The maximum growth rate was achieved at the optimal temperature, resulting in the highest cell count (Riskawati 2016). Following the description above, most AAB are mesophilic, with the optimal temperature for growth and acetic acid production recorded between 25°C and 30°C. The growth rate declines significantly at temperatures above 34°C. However, some thermotolerant strains were able to grow at temperatures ranging from 37°C to 45°C (El‐Askri et al. 2022; Hata et al. 2023; Hua et al. 2024; Kourouma et al. 2022). These strains often showed adaptation to high‐temperature conditions, common during food fermentation and storage (Kim et al. 2019). Arifuzzaman et al. (2014), reported that the genus *Acetobacter* could grow at temperatures between 25°C and 37°C, with the optimal temperature recorded at 30°C. Meanwhile, AAB observed to grow at 37°C was considered thermotolerant, as the majority were unable to develop above 34°C, which was regarded as the critical limiting factor in vinegar production (Wang et al. 2025).

#### 3.2.2 Catalase and Oxidase Tests

Catalase and oxidase tests were carried out to determine the bacteria's ability to produce catalase enzyme as well as detect the presence of cytochrome *c* oxidase, found in specific microorganisms (Sahiba et al. 2024). To ensure data reproducibility, catalase and oxidase assays were performed in triplicate. *A. indonesiensis* UNPADCC 01‐5 exhibited strong catalase activity, characterized by immediate and vigorous gas bubble formation upon exposure to 3% H_2_O_2_. In contrast, the oxidase test yielded a consistently negative result, evidenced by the absence of a colour change to deep blue or purple within the standard reaction time.

These phenotypic traits are consistent with the established characteristics of the genus *Acetobacter* and *A. indonesiensis* , which are typically catalase‐positive and oxidase‐negative (Ester 2022; Lisdiyanti et al. 2000). Beyond taxonomic identification, the robust catalase activity observed in strain UNPADCC 01‐5 suggests a critical functional adaptation to the specific ecological niche of oncom merah. As oncom merah undergoes solid‐state fermentation driven by the mould *Neurospora sitophila* under aerobic conditions, the associated microbiota is continuously exposed to ROS (Wijaya et al. 2024; Zandi and Schnug 2022). The production of catalase serves as an essential antioxidative defence mechanism, enabling the strain to neutralize toxic hydrogen peroxide generated during aerobic respiration, thereby maintaining cellular integrity and ensuring survival within the oxidative environment of the fermentation ecosystem (Dewi et al. 2022; Pulungan and Tumangger 2018).

#### 3.2.3 Antibiotic Sensitivity Test

Antibiotic susceptibility testing was conducted on *A. indonesiensis* UNPADCC 01‐5 using the Kirby‐Bauer method to determine the bacteria's sensitivity to clinically important antibiotics. Regarding the analysis, the antibiotics diffused into the MHA medium, inhibiting the growth of the test microorganism cultured on the agar. The results of the susceptibility testing for *A. indonesiensis* UNPADCC 01‐5 against several antibiotics are shown in Table 3.

**TABLE 3:** Inhibition zone of *A. indonesiensis* UNPADCC 01‐5 against antibiotics (mean ± SD, *n* = 3).

Antibiotics	Inhibition zone (mm)	Cell resistance to antibiotics	Antibiotic mechanisms
Erythromycin	21.04 ± 1.34	Susceptible	Inhibitors of bacterial protein synthesis by binding to the 50S ribosomal subunit (Kim et al. 2024).
Tetracycline	36.38 ± 1.38	Susceptible	Inhibitors of antibiotic protein synthesis by binding to the 30S ribosomal subunit (Kim et al. 2024).
Amoxicillin	17.28 ± 2.79	Intermediate	Inhibitors of bacterial cell wall synthesis (Verma et al. 2022).
Chloramphenicol	0 ± 0.0	Resistant	Inhibitors of bacterial protein synthesis by binding to the 50S ribosomal subunit (Verma et al. 2022).

*Note:* Resistant (inhibition zone ≤ 14 mm); intermediate (15–19 mm); susceptible (≥ 20 mm).

Antibiotic susceptibility testing revealed that *A. indonesiensis* UNPADCC 01‐5 is susceptible to tetracycline and erythromycin, intermediate to amoxicillin and resistant to chloramphenicol. These findings corroborate existing literature describing *A. indonesiensis* as typically resistant to chloramphenicol while remaining susceptible to tetracycline (Basu et al. 2018; Cepec and Trček 2022; Kohlmann et al. 2016). The observed chloramphenicol resistance is supported by the genomic identification of specific resistance determinants found in this study. The detection of the *mdtO* gene, which encodes a component of the Resistance‐Nodulation‐Division (RND) family efflux pumps, suggests a mechanism for the active extrusion of chloramphenicol (Elshobary et al. 2025; Rouvier et al. 2025). Additionally, the presence of the *ompA* gene, encoding a major outer membrane porin, likely contributes to reduced membrane permeability (Oh et al. 2025; Zhou et al. 2023). The synergistic action of reduced uptake via *OmpA* and active efflux via *MdtO* provides a plausible molecular basis for the strain's specific resistance to chloramphenicol (Belay et al. 2024; Rahmat Ullah et al. 2024). Regarding the *qacJ* gene, the detected sequence showed only weak similarity (41.35%), which falls below the threshold for confident functional annotation. Therefore, its contribution to the resistome cannot be definitively asserted without further experimental validation. We also acknowledge that while strain identity was established via 16S rRNA and biochemical profiling, whole‐genome phylogenomic metrics (such as ANI or dDDH) were not performed, representing a limitation in the definitive taxonomic assignment of the isolate.

### 3.3 Biochemical Characteristics

#### 3.3.1 Biochemical Tests

Biochemical or metabolic activities, associated with various chemical reactions within a living organism, played critical roles in the survival process. A comprehensive panel of biochemical assays was performed in duplicate (*n* = 2) on *A. indonesiensis* UNPADCC 01‐5 following standardized protocols (Cappuccino and Welsh 2018), with results summarized in Table 4.

**TABLE 4:** Biochemical characteristics of *A. indonesiensis* UNPADCC 01‐5.

Biochemical characteristics	Result
Arginine dihydrolase	−
Lysine decarboxylase	−
Ornithine decarboxylase	−
Utilization of citrate	+
Hydrogen sulfide production	−
Urease	−
Voges–Proskauer	−
Gelatinase	−
Glucose fermentation	+
Mannose fermentation	−
Inositol fermentation	−
Sorbitol Fermentation	−
Rhamnose fermentation	+
Sucrose fermentation	−
Melibiose fermentation	+
Amygdalin fermentation	−
Arabinose fermentation	+
ONPG	−

*Note:* + = positive; − = negative.

The biochemical characterization of *A. indonesiensis* UNPADCC 01‐5 revealed distinct metabolic capabilities relevant to its adaptation in the oncom merah fermentation environment. The isolate tested positive for citrate utilization, indicating the presence of citrate permease enzyme that enables citrate uptake and metabolism as a sole carbon source. Conversely, the strain showed negative results for arginine dihydrolase, lysine decarboxylase and ornithine decarboxylase tests. These enzymes typically function under anaerobic or microaerobic conditions, and their absence is consistent with previous reports regarding the family Acetobacteraceae and the obligate aerobic nature of *Acetobacter* species (Ester 2022; Heo et al. 2023).

An interesting discrepancy was observed in the Voges–Proskauer (VP) test results. The standard VP biochemical test yielded a negative reaction, indicating no detectable acetoin production under these conditions. However, GC–MS analysis of 5‐day fermentation cultures revealed acetoin as the dominant metabolite (73.37% relative abundance). This apparent contradiction can be attributed to the longer incubation duration used in the GC–MS analysis, which allowed for the accumulation of secondary metabolites that were undetectable during the shorter incubation period of the standard biochemical test (Harwoko et al. 2024). Acetoin production is metabolically significant as it contributes to the flavour profile, often described as having a buttery taste (Wang et al. 2025).

The gelatinase test demonstrated that *A. indonesiensis* UNPADCC 01‐5 lacks extracellular proteolytic activity, as evidenced by the absence of gelatin liquefaction. This characteristic is typical of the genus Acetobacter (Heo et al. 2023). Carbohydrate fermentation profiling revealed substrate specificity patterns relevant to the environment. The isolate successfully fermented glucose, rhamnose, melibiose and arabinose, while showing no fermentation of mannitol, inositol, sorbitol, sucrose and amygdalin. The observed fermentation profile of *A. indonesiensis* UNPADCC 01‐5 shows partial agreement with previous reports on this species. Lisdiyanti et al. (2000) reported that *A. indonesiensis* type strain could not grow on D‐mannitol but showed positive D‐glucose fermentation, consistent with our findings. Furthermore, Arifuzzaman et al. (2014) noted that *Acetobacter* spp. are generally able to utilize glucose, arabinose and melibiose, but are unable to use sucrose, mannitol and sorbitol.

The observed biochemical profile reflects the strain's metabolic adaptation to the oncom merah fermentation environment, which is made from tofu processing waste, cassava pulp and peanut press cake (Aini et al. 2020; Wikanta 2019). The ability to ferment specific carbohydrates suggests adaptation to these plant‐derived substrates. These metabolic characteristics, combined with its acid production capability, suggest a potential role as a functional adjunct culture.

### 3.4 Analysis of Organic Compounds

Organic compounds are the main components that influence the volatility and flavour of fruit vinegar. Changes in the volatile components of fruit vinegar were connected to a series of chemical reactions and microbial metabolism during fermentation and aging (Li et al. 2023). Based on the analysis, fermentation led to the synthesis or breakdown of compounds with sensory properties (e.g., sugars, amino acids and organic acids, including alcohols), depending on certain conditions such as temperature, starter culture and incubation time (Gil et al. 2020). The analysis of volatile compounds included alcohols, acids, esters, ketones and aldehydes (Wang et al. 2025).

The results of the GC–MS chromatogram (Data S2) obtained from *A. indonesiensis* UNPADCC 01‐5, incubated for 5 days, showed the evolution of several compounds at various retention times (RT). A total of 13 compounds were successfully identified, as shown in Table 5.

**TABLE 5:** Compound components of *A. indonesiensis* UNPADCC 01‐5 detected by GC–MS.

No	Compound	RT (min)	Peak height	Area	Compound groups
1	Acetoin	4.856	11135.44	32891.88	Ketone
2	Acetic acid	6.671	3358.66	8931.01	Organic acids
3	2‐Butanol, 3‐methyl—	6.05	412.96	3040.21	Alcohol
4	2‐Piperidinone	17.619	1273.6	1584.49	Cyclic compounds containing nitrogen
5	DL‐2,3‐Butanediol	20.808	280.6	1252.61	Secondary alcohol resulting from the reduction of acetone
6	3‐Hexanol, 2,4‐dimethyl—	2.3	328.06	1156.85	Branched‐chain alcohols
7	L‐Serine, ethyl ester	11.228	223.64	671.19	Esters of amino acids
8	Boronic acid, ethyl—	16.49	175.99	667.21	Organoboron compounds
9	1‐Methyl‐2‐propenylhydrazine	9.469	144.49	589.01	Volatile nitrogen‐based compounds, hydrazine derivatives
10	Acetic acid, hydrazide	8.789	145.39	380.19	Nitrogen‐containing derivatives of acetic acid
11	Propanoic acid, 2‐(aminooxy)—	20.813	155.93	354.05	Amino acid derivatives
12	Carbonyl sulfide	10.202	157.35	352.93	Volatile sulfur compounds
13	Serine	11.215	165.35	333.96	Amino acid

The analysis revealed that the volatile profile was dominated by ketones and organic acids. Acetoin (3‐hydroxy‐2‐butanone) was the most abundant compound, with a relative peak area of 73.37%, followed by acetic acid at 27.15%. The high production of acetoin is biologically relevant, as *Acetobacter* species are known to oxidize lactic acid into acetoin and acetic acid during fermentation (Lu et al. 2016). Acetoin acts as a significant flavour compound, contributing a pleasant creamy, buttery, or yogurt‐like aroma that enhances the sensory quality of fermented products (Wang et al. 2025).

Acetic acid was the second major metabolite identified. This finding is consistent with the metabolic characteristics of AAB, specifically the genus *Acetobacter*, which plays a significant role in acetic acid metabolism primarily through the acetyl‐CoA, acetyl‐adenylate and acetaldehyde pathways (Li et al. 2023). The presence of acetic acid is supported by the WGS data, which explicitly identified genes associated with acetic acid production. Beyond its role in flavour, acetic acid exhibits antimicrobial properties that inhibit pathogenic bacteria in the digestive tract and contributes to health benefits such as blood glucose regulation (Claudia et al. 2022). Furthermore, it has been reported to have beneficial effects on hypertension, hyperglycaemia, dyslipidaemia, obesity and hypercholesterolemia (Hata et al. 2023; Li et al. 2023).

Several minor compounds were also detected, including 2,3‐butanediol, which is formed from the reduction of acetoin and possesses antiseptic properties, with a 0.1% solution exhibiting a bactericidal effect against many pathogenic bacteria (Cesselin et al. 2021; Petrov and Petrova 2021). Amino acid derivatives such as serine were present; serine is known to add sweetness to the flavour profile of fermented vinegars (Wang et al. 2025). However, the detection of certain compounds, specifically boronic acid, ethyl‐ and nitrogen‐containing hydrazine derivatives (Table 5), requires cautious interpretation. These compounds are atypical for Acetobacter fermentation and likely represent analytical artefacts or non‐metabolic derivatives from the growth medium rather than direct bacterial metabolites.

### 3.5 Genetic Characteristics

**FIGURE 2:**
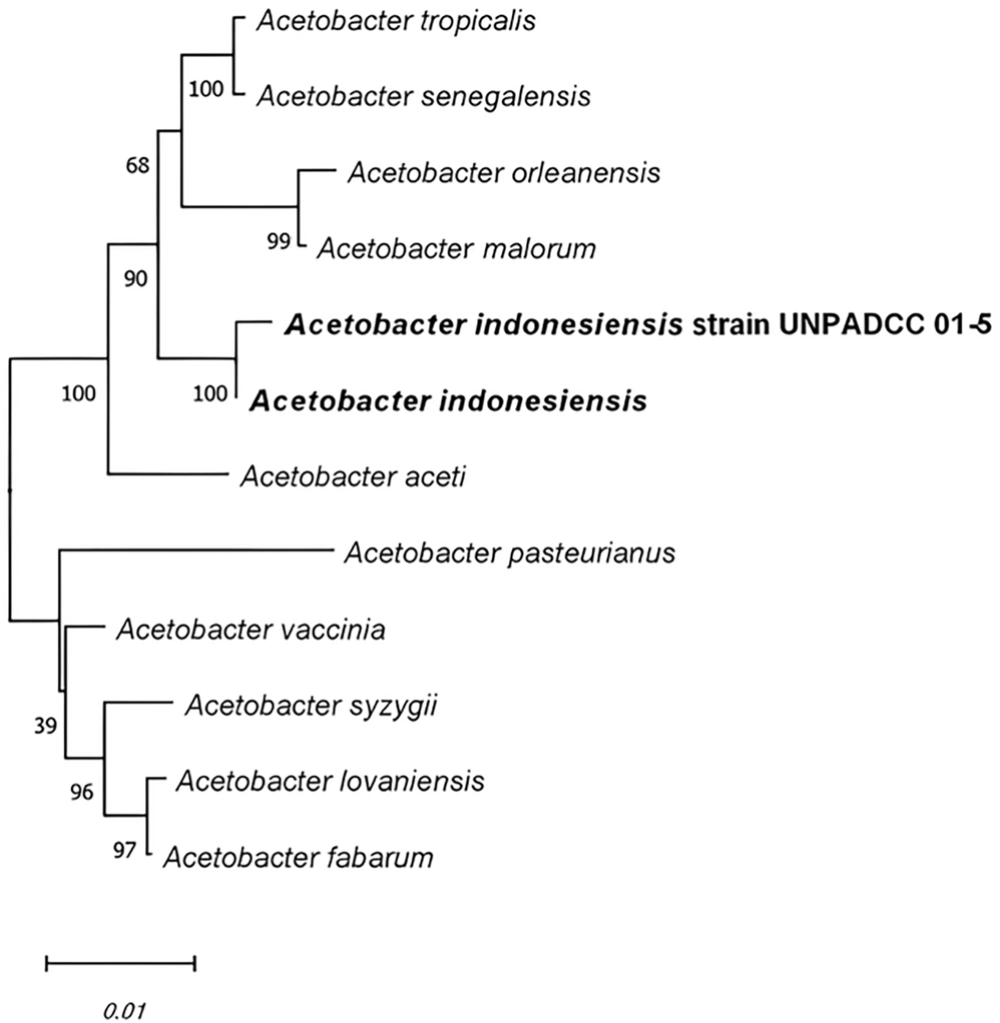
Phylogenetic tree construction of *A. indonesiensis* UNPADCC 01‐5 Based on 16S rRNA (accession number: PV660664).

Molecular identification was performed by conducting WGS. The results obtained based on the annotated genome of the bacterial isolate using a fragment of the 16S rRNA gene sequence showed that the isolate matched with *A. indonesiensis* as in Figure 2. The 16S rRNA gene sequence (1486 bp) of UNPADCC 01‐5 was deposited in GenBank with accession number PV660664 (https://www.ncbi.nlm.nih.gov/nuccore/PV660664). Meanwhile, the WGS of UNPADCC 01‐5 had the accession number JBPULA010000000 and was registered at the Universitas Padjadjaran Culture Collection (UNPADCC, https://ccinfo.wdcm.org/details?regnum=1330). Phylogenetic trees reflected the evolutionary relationships between species or gene families and facilitated identification (Zou et al. 2024). Figure 2 shows the phylogenetic tree for the strain UNPADCC 01‐5, constructed according to the 16S rRNA gene sequence using the neighbour‐joining method with 1000 bootstrap replications.

The phylogenetic analysis, in line with the 16S rRNA sequence, showed that the bacteria belonged to the genus *Acetobacter*. The analysed isolate, strain UNPADCC 01‐5, exhibited a high sequence similarity with *A. indonesiensis* . The isolate was grouped under the same clade with strong bootstrap (100%), representing a close taxonomic relationship. Further insights into the functional information of the strain were provided by WGS data and comparative genomic analysis. The results of the WGS annotation in DFAST are shown in Table 6.

**TABLE 6:** Genome statistics data of *A. indonesiensis* UNPADCC 01‐5.

Genome statistics data	
Genome size (bp)	3,420,477
Number of sequences	164
Content value G+C	54.1%
Number of CRISPR	2
Number of tRNA	44
Number of rRNA	3
Number of CDS	3,079

*A. indonesiensis* UNPADCC 01‐5 has a total genome size of 3.4 Mbp with 164 contigs and 3079 coding sequences (CDS). The G+C content of the bacterial sample is 54.1%, with rapid Annotations Subsystems Technology (RAST) used to explain the *A. indonesiensis* UNPADCC 01‐5 draft genome. Data S3 shows certain general features of the genes, as derived from the RAST analysis.

Screening for virulence and resistance determinants using VFDB, CARD, ResFinder and PlasmidFinder did not detect any genes associated with human virulence factors or pathogenic secretion systems. The results of the detected target genes, including probiotic, bioremediation, antioxidant and potential plant growth‐promoting bacteria (PGPB) properties, were shown in the genomic circle diagram in Data S4 and S5. Moreover, microbial biotechnology played a crucial role in producing significant natural bioactive products, essential for advancements in genetic engineering, functional genomics and synthetic biology (Santos‐Beneit 2024). These gene‐based technologies were applied in various fields, such as health, food and the environment.

Extending these observations, this study provides specific insights into the functional role of *A. indonesiensis* UNPADCC 01‐5 within the oncom merah fermentation system. The strain's capacity to produce acetic acid and acetoin indicates an active role in carbon metabolism, particularly in substrate transformation and energy generation under fermentation conditions. These metabolic activities directly influence the surrounding environment through acidification, which in turn shapes microbial community structure by suppressing less tolerant or competing microorganisms while promoting acid‐adapted populations. Such interactions highlight the dynamic relationship between microbial activity and environmental conditions in traditional fermentation systems. Importantly, as the first report of *A. indonesiensis* from oncom merah, this study expands current knowledge of microbial diversity and provides mechanistic insight into how indigenous bacteria contribute to ecosystem stability and function. Collectively, these findings offer a clearer understanding of microbe–environment interactions and demonstrate the ecological and functional relevance of this species in fermented food systems.

## 4 Conclusion

*Acetobacter indonesiensis* UNPADCC 01‐5 isolated from oncom merah demonstrated tolerance to acidic pH, moderate salinity and elevated temperatures, traits that are consistent with previously reported characteristics of *A. indonesiensis* . In the absence of direct comparative analyses with other isolates, these physiological properties should be interpreted as indicative of environmental adaptability rather than as species‐unique traits. Biochemical profiling confirmed the strain's ability to ferment multiple carbohydrates, including glucose, rhamnose, melibiose and arabinose. GC–MS analysis revealed acetoin as the dominant metabolite, followed by acetic acid, highlighting its potential relevance for flavour development in acidic fermentation systems. Antibiotic susceptibility testing showed selective resistance to chloramphenicol and intermediate resistance to amoxicillin. These phenotypic results were supported by genomic identification of antimicrobial resistance–associated genes (e.g., *ompA* and *mdtO*), demonstrating concordance between phenotypic and genotypic analyses and underscoring the value of an integrated characterization approach. Screening for virulence and resistance determinants did not detect any genes associated with human virulence factors or pathogenic secretion systems. Overall, this study establishes the physiological robustness, metabolic capability and genomic features of *A. indonesiensis* UNPADCC 01‐5 as a promising candidate for use as an adjunct culture in acidic fermentation processes. However, claims regarding probiotic functionality or environmental bioremediation potential remain preliminary. Further studies are required, including in vivo probiotic validation, phylogenomic confirmation using ANI and dDDH analyses, optimization of acetoin and acetic acid production under controlled fermentation conditions and functional validation of key genes through transcriptomic or targeted genetic approaches.

## Author Contributions

**Diffa Amanda Iswhara:** investigation, writing – review and editing, software, validation. **Rianti Nur Azzahra:** visualization, investigation, data curation, software. **Annisa:** writing – review and editing, data curation, supervision. **Felice Olivia Lengkey:** investigation, writing – review and editing, validation. **Erisa Zahra:** writing – original draft, writing – review and editing, investigation, data curation, visualization, software. **Syiffa Fauzia:** writing – review and editing, formal analysis. **Xiwu Jia:** writing – review and editing, formal analysis. **Ratu Safitri:** writing – review and editing, formal analysis. **Yolani Syaputri:** writing – original draft, writing – review and editing, conceptualization, methodology, supervision, visualization, funding acquisition. **Asri Peni Wulandari:** writing – review and editing, formal analysis.

## Funding

The authors express their gratitude to Universitas Padjadjaran for funding this research through Hibah Internal Riset Kompetensi Dosen Unpad (RKDU) (Contract No. 5870/UN6.D/PT.00/2026) awarded to Yolani Syaputri and this publication charge is funded by Unpad through the Indonesian Endowment Fund for Education (LPDP) on behalf of the Indonesian Ministry of Higher Education, Science and Technology and managed under the EQUITY Program (Contract No. 4303/B3/DT.03.08/2025 and 3927/UN6. RKT/HK.07.00/2025).

## Ethics Statement

The authors have nothing to report.

## Consent

The authors have nothing to report.

## Conflicts of Interest

The authors declare no conflicts of interest.

## Supporting information


**Data S1:** Morphological characterization of *A. indonesiensis* UNPADCC 01‐5. (A) Morphological macroscopic colony; (B) Gram staining profile; (C) Morphological cell and dimensions.
**Data S2:** GC–MS chromatogram of the fermentation product of *A. indonesiensis* UNPADCC 01‐5 after 5 days.
**Data S3:** Gene annotation results in RAST.
**Data S4:** Genome diagram of *A. indonesiensis* UNPADCC 01‐5.
**Data S5:** Selected genes from genome diagram visualization results of *A. indonesiensis* UNPADCC 01‐5.

## Data Availability

The data that support the results of this study are available from the corresponding author upon reasonable request.
