# Supplementary material for: Polyphasic Characterization of Acetobacter indonesiensis UNPADCC 01‐5 Isolated From a Traditional Fermented Food, Oncom Merah
Source: Environ Microbiol Rep. 2026 Jun 4;18(3):e70346. doi: 10.1111/1758-2229.70346 (PMC13238706; doi:10.1111/1758-2229.70346)
Supplement: Supplementary file 1 — Data S1: Morphological characterization of A. indonesiensis UNPADCC 01‐5. (A) Morphological macroscopic colony; (B) Gram staining profile; (C) Morphological cell and dimensions. Data S2: GC–MS chromatogram of the fermentation product of A. indonesiensis UNPADCC 01‐5 after 5 days. Data S3: Gene annotation results in RAST. Data S4: Genome diagram of A. indonesiensis UNPADCC 01‐5. Data S5: Selected genes from genome diagram visualization results of A. indonesiensis UNPADCC 01‐5. [file EMI4-18-e70346-s001.docx]

**Supplementary Data 1**. Morphological characterization of A. indonesiensis UNPADCC 01-5. (A) Morphological macroscopic colony; (B) Gram staining profile; (C) Morphological cell and dimensions.


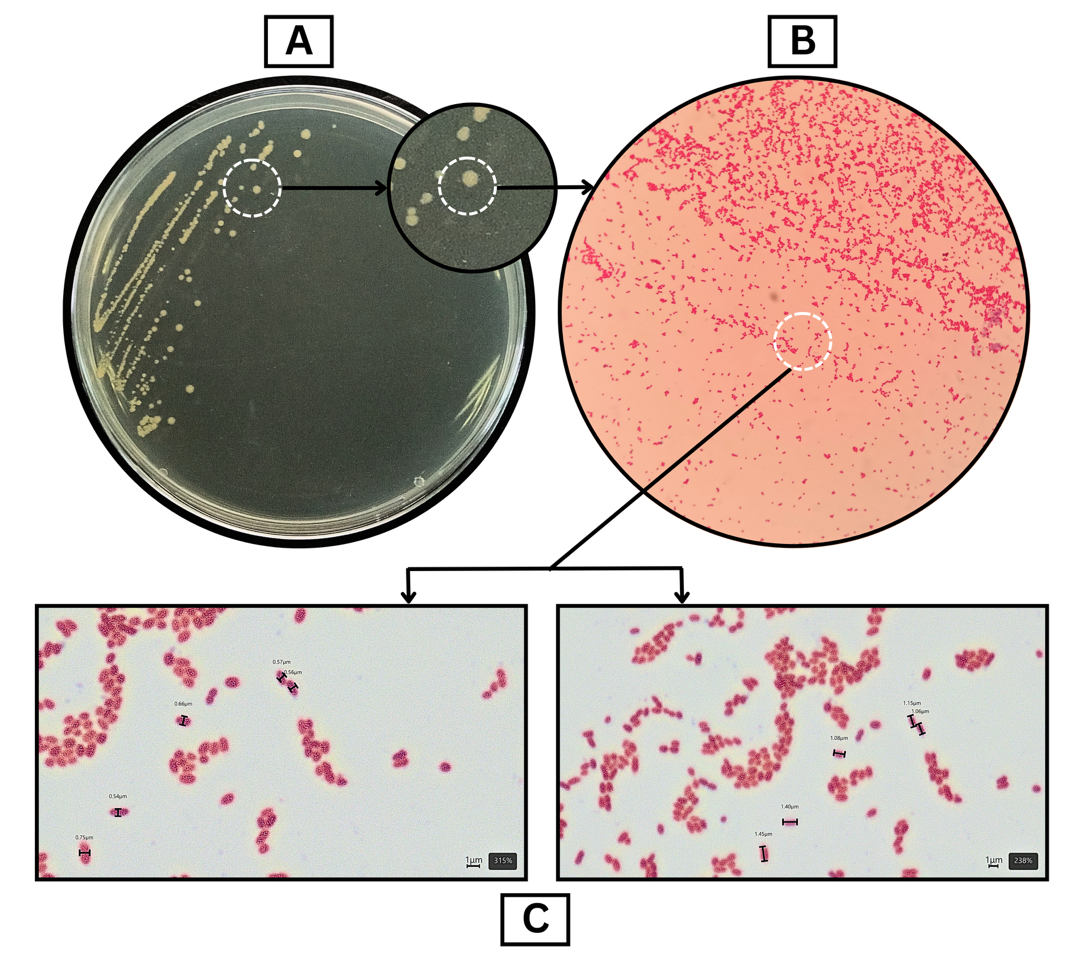


**Supplementary Data 2.** GC-MS chromatogram of the fermentation product of A. indonesiensis UNPADCC 01-5 after 5 days


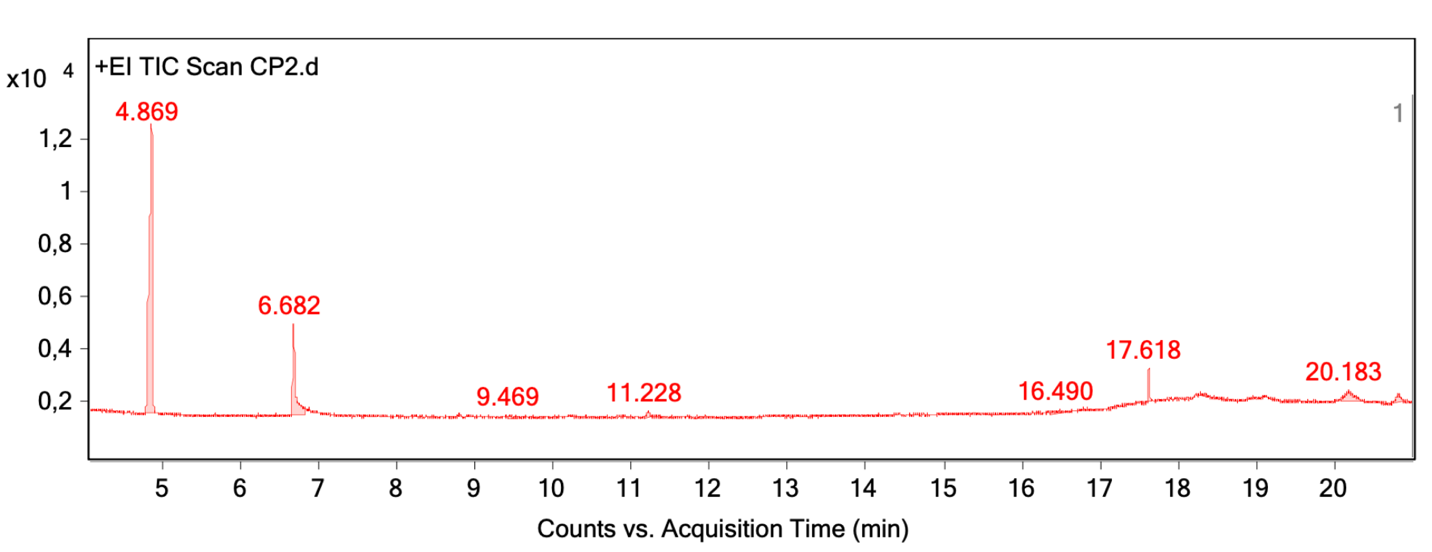


**Supplementary Data 3.** Gene Annotation Results in RAST


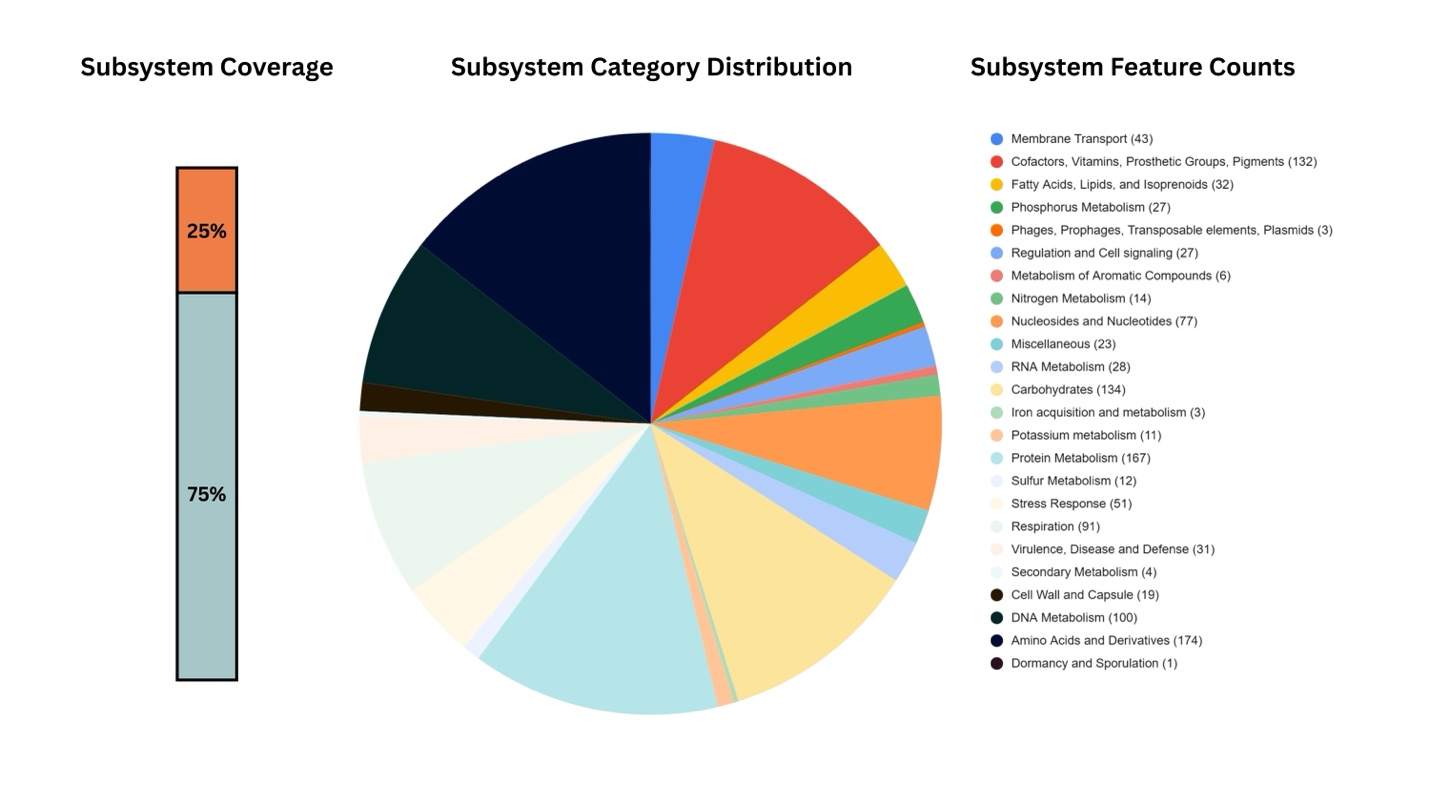


**Supplementary Data 4.** Genome Diagram of *A. indonesiensis* UNPADCC 01-5


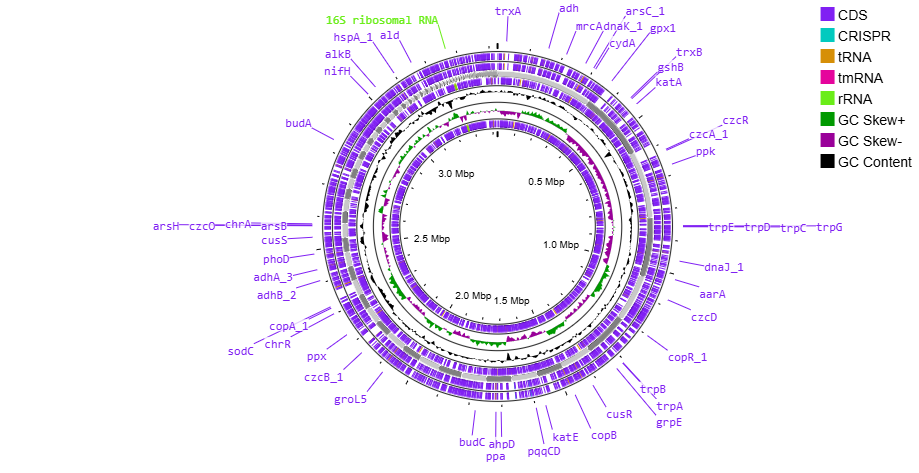


**Supplementary Data 5**. Selected Genes from Genome Diagram Visualization Results of A. indonesiensis UNPADCC 01-5

| **Genes** | **Functions** | **Purpose** | **References** |
| --- | --- | --- | --- |
| *budA, budC* | α-acetolactate decarboxylase; meso-2,3-BD dehydrogenase | The *budA* gene encodes alpha-acetolactate decarboxylase, an enzyme that converts acetolactate to acetoin, as well as regulates internal pH within cells. The *budC* gene converts acetoin to 2,3-butanediol. | (Lu et al., 2016) |
| *adh, ald, pqqCD* | Alcohol dehydrogenase gene, aldehyde dehidrogenase pyrroloquinoline quinone synthesis genes | This isolate plays a role in the oxidation of ethanol to acetic acid in vinegar production. *Acetobacter* uses two membrane-bound enzymes (Alcohol Dehydrogenase (ADH) and Aldehyde Dehydrogenase (ALDH)) to oxidize ethanol to acetic acid during respiration. It further oxidizes acetic and lactic acid to carbon dioxide and water. However, membrane-bound ADH (PQQ-ADH) is a major enzyme in acetic acid oxidative fermentation, playing an important role in enhancing the acid tolerance of AAB. ALDH encodes the gene that oxidizes acetaldehyde into acetic acid. | (Hua et al., 2024; Qiu et al., 2021; Zhang et al., 2023). |
| *Hsp* | Heat shock protein | When bacteria are subjected to various environmental stresses, *hsp* is activated. Additionally, *hsp* promotes protein homeostasis and cell fitness. | (Zhang et al., 2023) |
| *trxA, trxB* | Thioredoksin (trx)-reduktase | The bacterial *trx* system plays an important role in various major processes. This included stress response, metabolism, regulatory cascades, motility, biofilm formation, and virulence. | (Anjou et al., 2024) |
| *dnaK, dnaJ, grpE, groL, groS* | Chaperon genes | The genes associated with BAA thermotolerance are mostly included in the heat shock protein family, namely *dnaK, dnaJ, grpE, groES*, and *groEL*. These tend to increase the heat tolerance of the strain as well as its resistance to acetic acid. | (Hua et al., 2024) |
| *aarA* | Acetic acid resistance gene | This gene increases acetate tolerance in *Acetobacter.* | (Hua et al., 2024) |
| *katA, katE, sod, gpx1, gshB,* | Oxidative stress response genes | In response to intracellular oxidative stress caused by the accumulation of reactive oxygen species (ROS), cells tend to activate its detoxification mechanisms, consisting of enzymatic and non-enzymatic defense systems. The enzymatic system comprised superoxide dismutase (SOD), catalase (CAT), and glutathione peroxidase (GPx). Meanwhile, the non-enzymatic system mainly included glutathione (GSH) and vitamins C/E. Cost-effective antioxidant supplementation, such as ascorbic acid, combined with the overexpression of ROS-scavenging enzymes (such as SodA and KatE), significantly enhanced porphyrin biosynthesis, outlining its potential for industrial-scale applications. | (Arab et al., 2025; Li et al., 2023) |
| *ahpD* | Alkyl hydroperoxidase, sistem enzim antioksidan | Alkyl hydroperoxidase activity provides important antioxidant defense for bacterial cells. The catalytic mechanism requires two peroxidases, *AhpC* and *AhpD*, in which *AhpD* acts as an important adapter protein. | (Clarke et al., 2011) |
| *nifH* | Nitrogenase reductase | Gene expression produced by nitrogen-fixing bacteria that play an important role alongside *Arbuscular mycorrhizal* fungi in maintaining plant growth and soil function. | (Liang et al., 2023) |
| *trpA, trpB, trpC, trpD, trpE, trpF, trpG, trpS* | Tryptophan operon | Indole-3-acetic acid (IAA) is a phytohormone that functions as an auxin in plants and influences various physiological processes. In one of the IAA biosynthetic pathways, anthraquinone acid (AA) promoted corn plant growth, participated in the phytohormone synthesis pathway, and exhibited bacteriostatic activity against phytopathogens. AA acted as an intermediate metabolite in the synthesis and degradation of tryptophan. The *trp* operon enabled the metabolism of essential amino acid such as tryptophan. The enzymes associated with the conversion of koresmat through AA into tryptophan were components of anthranilate synthase I and II (*TrpE* and *TrpD*), indole-3-glycerol phosphate synthase/N(-5-phosphoribosyl) anthranilate isomerase (*TrpC*), including the β and α subunits of tryptophan synthase (*TrpB* and *TrpA*). | (Vega-Celedón et al., 2024) |
| *ppa, ppk, ppx, phoD* | Inorganic pyrophosphatase, polyphosphate kinase, exopolyphosphatase, alkaline phosphatase | The *ppa, ppk,* and *ppx* are inorganic phospholytic genes that encode inorganic and extra pyrophosphatase, as well as polyphosphate kinase. These genes were found in polyphosphate-accumulating or phosphorus-solubilizing bacteria that played a role in enhanced biological phosphate removal and heavy metal uptake. Under conditions of inorganic phosphorus deficiency, *phoA* and *phoD* are the most common genes. Additionally, *phoD* was the strongest associated with alkaline phosphatase by phosphorus-solubilizing bacteria. | (Pan & Cai, 2023; Srivastava et al., 2022; Tao et al., 2024) |
| *alkB* | Alkane hydroxylase/  alkanmonooxygenase | The gene (*alkB*) is associated with the most efficient hydrocarbon-degrading bacteria. *AlkB* is highly diverse but shares common features related to the aerobic degradation of petroleum n-alkanes and products. The *Alk* system is key to the synthesis of polymers relevant to pharmacy and medicine. Alkane monooxygenase (*AlkB*), a membrane-covering metalloenzyme, converts straight-chain alkanes into alcohols in the initial step of microbial-mediated alkane degradation, thereby playing a relevant role in the global carbon cycle and oil bioremediation. | (Fenibo et al., 2023; Guo et al., 2023; Shapiro et al., 2022) |
| *copA, copB, copR, copZ* | Copper resistance gene cluster | The *cop* gene is crucial for bacterial copper homeostasis, enabling resistance to copper toxicity and contributing to virulence in pathogens. The structural features of the *cop* operon provided a general regulatory mechanism that coordinated the response to copper exposure within the cell. Furthermore, two subfamilies of ATPases, *CopA* and *CopB*, are major players in cellular copper export. *CopA* and *CopB* transports Cu^+^, and transports Cu^2+^, respectively. Metallochaperones from the *CopZ* family converts Cu^2+^ to Cu^+^ and were associated with transporting reduced ions to *CopA. CopR* acts as a regulator to assist *CopB* in transporting copper ions. According to (Y. Wang et al., 2023), copper stabilizes yogurt acidity and enhances the flavor through the *cop* gene in *Lactobacillus delbrueckii* ssp. *Bulgaricus* ATCC11842, which effectively inhibits yogurt post-acidification after its gene expression is regulated. | (Ge et al., 2021; Grünberger et al., 2020; Sullivan et al., 2021; Y. Wang et al., 2023) |
| *cusS, cusR* | Copper-responsive two-component system | Copper tolerance genes such as *cop*. *CusS-CusR* increases resistance to Cu, while reducing virulence. The CUS was initially identified as a silver resistance system. It was found to be connected to anaerobic copper homeostasis due to the similar chemical properties with silver and copper. This system was responsible for transporting copper/silver ions into the extracellular space. | (Gudipaty & McEvoy, 2014; He et al., 2021) |
| *arsB, arsC, arsH* | Arsenic resistance genes | The *ars* operon plays an essential role in microbial arsenic resistance systems, which served as a template for designing synthetic bacteria. *arsABCD* belonged to the arsenic resistance gene. The *arsC* gene encoded arsenate reductase. The *ArsB* permease is an As (III) efflux pump that extruded trivalent As (III) from cells. The *arsH* gene encoded methylarsenite oxidase. | (Flores et al., 2022; Hui et al., 2024; Yang & Rosen, 2016) |
| *czcA,*  *czcB,*  *czcD,*  *czcR,*  *czcO* | Cobalt, zinc, and cadmium resistance genes | One of the cadmiums (Cd) resistance genes. The *CzcA, CzcB,* and *CzcC* proteins comprised an active efflux protein complex that pumped Cd^2+^ out of microbial cells. *CzcA* functioned as a cation/proton antiporter, *CzcB* as a cation-binding subunit, with *CzcC* modifying the substrate specificity to Cd²⁺, and *CzcD* coordinating the expression control of the *czc* gene cluster, functioning as an extracellular cation sensor. Therefore, appropriate expression levels of the *czc* genes ensured that the cell was sufficiently resistant to heavy metals. | (M. Wang et al., 2021) |
| *chrR,*  *chrA* | Chromium resistence gene | *ChrA* is a well-known anti-chromium gene that belonged to the chromate ion transporter. Intracellular chromium was pumped out of the cell through the action of the *chrA* protein, thereby increasing the strain's tolerance to chromium. Chromate reductase (*ChrR*), a bacterial enzyme from *Pseudomonas putida* was used in bioremediation to remove chromate from the environment. This was realized in a cost-effective and environmentally safe manner. *ChrR* reduced Cr(VI) to Cr(III). A comprehensive analysis of the *ChrR* sequence showed its functions in metal ion binding, iron-sulfur cluster binding, oxidoreductase, and catalytic activities. | (Su et al., 2022; Tasleem et al., 2022) |

**REFERENCES**

​​Anjou, C., Lotoux, A., Morvan, C., & Martin‐Verstraete, I. (2024). From Ubiquity to Specificity: The Diverse Functions of Bacterial Thioredoxin Systems. *Environmental Microbiology*, *26*(6). <https://doi.org/10.1111/1462-2920.16668>

​Arab, B., Moo-Young, M., Liu, Y., & Chou, C. P. (2025). Manipulating Intracellular Oxidative Conditions to Enhance Porphyrin Production in Escherichia coli. *Bioengineering*, *12*(1), 83. <https://doi.org/10.3390/bioengineering12010083>

​Clarke, T. E., Romanov, V., Chirgadze, Y. N., Klomsiri, C., Kisselman, G., Wu-Brown, J., Poole, L. B., Pai, E. F., & Chirgadze, N. Y. (2011). Crystal Structure of Alkyl Hydroperoxidase D Like Protein PA0269 From Pseudomonas aeruginosa: Homology of the AhpD-Like Structural Family. *BMC Structural Biology*, *11*(1), 27. <https://doi.org/10.1186/1472-6807-11-27>

​Fenibo, E. O., Selvarajan, R., Abia, A. L. K., & Matambo, T. (2023). Medium-Chain Alkane Biodegradation and Its Link to Some Unifying Attributes of alkB Genes Diversity. *Science of The Total Environment*, *877*, 162951. <https://doi.org/10.1016/j.scitotenv.2023.162951>

​Flores, A., Valencia-Marín, M. F., Chávez-Avila, S., Ramírez-Díaz, M. I., de los Santos-Villalobos, S., Meza-Carmen, V., Orozco-Mosqueda, Ma. del C., & Santoyo, G. (2022). Genome Mining, Phylogenetic, and Functional Analysis of Arsenic (As) Resistance Operons in Bacillus Strains, Isolated From As-Rich Hot Spring Microbial Mats. *Microbiological Research*, *264*, 127158. <https://doi.org/10.1016/j.micres.2022.127158>

​Ge, Q., Cobine, P. A., & De La Fuente, L. (2021). The Influence of Copper Homeostasis Genes *copA* and *copB* on *Xylella fastidiosa* Virulence Is Affected by Sap Copper Concentration. *Phytopathology®*, *111*(9), 1520–1529. <https://doi.org/10.1094/PHYTO-12-20-0531-R>

​Grünberger, F., Reichelt, R., Waege, I., Ned, V., Bronner, K., Kaljanac, M., Weber, N., El Ahmad, Z., Knauss, L., Madej, M. G., Ziegler, C., Grohmann, D., & Hausner, W. (2020). *CopR, a Global Regulator of Transcription to Maintain Copper Homeostasis in Pyrococcus furiosus*. <https://doi.org/10.1101/2020.08.14.251413>

​Gudipaty, S. A., & McEvoy, M. M. (2014). The Histidine Kinase CusS Senses Silver Ions Through Direct Binding by Its Sensor Domain. *Biochimica et Biophysica Acta (BBA) - Proteins and Proteomics*, *1844*(9), 1656–1661. <https://doi.org/10.1016/j.bbapap.2014.06.001>

​Guo, X., Zhang, J., Han, L., Lee, J., Williams, S. C., Forsberg, A., Xu, Y., Austin, R. N., & Feng, L. (2023). Structure and Mechanism of the Alkane-Oxidizing Enzyme AlkB. *Nature Communications*, *14*(1), 2180. <https://doi.org/10.1038/s41467-023-37869-z>

​He, R., Zuo, Y., Zhao, L., Ma, Y., Yan, Q., & Huang, L. (2021). Copper Stress by Nutritional Immunity Activates the CusS-CusR Two-Component System That Contributes to Vibrio alginolyticus Anti-Host Response but Affects Virulence-Related Properties. *Aquaculture*, *532*, 736012. <https://doi.org/10.1016/j.aquaculture.2020.736012>

​Hua, S., Wang, Y., Wang, L., Zhou, Q., Li, Z., Liu, P., Wang, K., Zhu, Y., Han, D., & Yu, Y. (2024). Regulatory Mechanisms of Acetic Acid, Ethanol, and High Temperature Tolerances of Acetic Acid Bacteria During Vinegar Production. *Microbial Cell Factories*, *23*(1), 324. <https://doi.org/10.1186/s12934-024-02602-y>

​Hui, C., Liu, M., & Guo, Y. (2024). Synthetic Bacteria Designed Using ars Operons: A Promising Solution for Arsenic Biosensing and Bioremediation. *World Journal of Microbiology and Biotechnology*, *40*(6), 192. <https://doi.org/10.1007/s11274-024-04001-2>

​Li, G., Yan, N., Li, G., & Wang, J. (2023). Optimization of the Process for Green Jujube Vinegar and Organic Acid and Volatile Compound Analysis during Brewing. *Foods*, *12*(17), 3168. <https://doi.org/10.3390/foods12173168>

​Liang, M., Wu, Y., Zhao, Q., Jiang, Y., Sun, W., Liu, G., Ma, L., & Xue, S. (2023). Secondary vegetation succession on the Loess Plateau altered the interaction between arbuscular mycorrhizal fungi and nitrogen-fixing bacteria. *Forest Ecology and Management*, *530*, 120744. <https://doi.org/10.1016/j.foreco.2022.120744>

​Lu, Z. M., Liu, N., Wang, L. J., Wu, L. H., Gong, J. S., Yu, Y. J., Li, G. Q., Shi, J. S., & Xu, Z. H. (2016). Elucidating and regulating the acetoin production role of microbial functional groups in multispecies acetic acid fermentation. *Applied and Environmental Microbiology*, *82*(19), 5860–5868. <https://doi.org/10.1128/AEM.01331-16>

​Pan, L., & Cai, B. (2023). Phosphate-Solubilizing Bacteria: Advances in Their Physiology, Molecular Mechanisms and Microbial Community Effects. *Microorganisms*, *11*(12), 2904. <https://doi.org/10.3390/microorganisms11122904>

​Qiu, X., Zhang, Y., & Hong, H. (2021). Classification of acetic acid bacteria and their acid resistant mechanism. *AMB Express*, *11*(1), 29. <https://doi.org/10.1186/s13568-021-01189-6>

​Shapiro, T. N., Manucharova, N. A., & Lobakova, E. S. (2022). Activity of alkanmonooxygenase &lt;i&gt;alk&lt;/i&gt;B gene in strains of hydrocarbon-oxidizing bacteria isolated from petroleum products. *Vavilov Journal of Genetics and Breeding*, *26*(6), 575–582. <https://doi.org/10.18699/VJGB-22-70>

​Srivastava, S., Anand, V., Kaur, J., Ranjan, M., Bist, V., Asif, M. H., & Srivastava, S. (2022). Functional Genetic Diversity and Plant Growth Promoting Potential of Polyphosphate Accumulating Bacteria in Soil. *Microbiology Spectrum*, *10*(1). <https://doi.org/10.1128/spectrum.00345-21>

​Su, Y., Sun, S., Liu, Q., Zhao, C., Li, L., Chen, S., Chen, H., Wang, Y., & Tang, F. (2022). Characterization of the simultaneous degradation of pyrene and removal of Cr(VI) by a bacteria consortium YH. *Science of The Total Environment*, *853*, 158388. <https://doi.org/10.1016/j.scitotenv.2022.158388>

​Sullivan, M. J., Goh, K. G. K., Gosling, D., Katupitiya, L., & Ulett, G. C. (2021). Copper Intoxication in Group B Streptococcus Triggers Transcriptional Activation of the *cop* Operon That Contributes to Enhanced Virulence during Acute Infection. *Journal of Bacteriology*, *203*(19). <https://doi.org/10.1128/JB.00315-21>

​Tao, A., Chen, Z., Wang, K., Wang, Z., Zhang, Y., Luo, X., Lu, Y., & Su, C. (2024). Effects of phosphorus sources on the transformation of phosphorus forms, microbial community, and functional genes in up-flow anaerobic sludge bed reactor. *Biochemical Engineering Journal*, *209*, 109382. <https://doi.org/10.1016/j.bej.2024.109382>

​Tasleem, M., El-Sayed, A.-A. A. A., Hussein, W. M., & Alrehaily, A. (2022). Bioremediation of Chromium-Contaminated Groundwater Using Chromate Reductase from Pseudomonas putida: An In Silico Approach. *Water*, *15*(1), 150. <https://doi.org/10.3390/w15010150>

​Vega-Celedón, P., Castillo-Novales, D., Bravo, G., Cárdenas, F., Romero-Silva, M. J., & Seeger, M. (2024). Synthesis and Degradation of the Phytohormone Indole-3-Acetic Acid by the Versatile Bacterium Paraburkholderia xenovorans LB400 and Its Growth Promotion of Nicotiana tabacum Plant. *Plants*, *13*(24), 3533. <https://doi.org/10.3390/plants13243533>

​Wang, M., Wang, L., Shi, H., Liu, Y., & Chen, S. (2021). Soil bacteria, genes, and metabolites stimulated during sulfur cycling and cadmium mobilization under sodium sulfate stress. *Environmental Research*, *201*, 111599. <https://doi.org/10.1016/j.envres.2021.111599>

​Wang, Y., Li, D., Chitrakar, B., Zhang, X., Zhang, N., Liu, C., Li, Y., Wang, M., Tian, H., & Li, C. (2023). Copper inhibits postacidification of yogurt and affects its flavor: A study based on the Cop operon. *Journal of Dairy Science*, *106*(2), 897–911. <https://doi.org/10.3168/jds.2022-22369>

​Yang, H.-C., & Rosen, B. P. (2016). New mechanisms of bacterial arsenic resistance. *Biomedical Journal*, *39*(1), 5–13. <https://doi.org/10.1016/j.bj.2015.08.003>

​Zhang, W., Weng, P., & Wu, Z. (2023). Interaction profile of a mixed-culture fermentation of Issatchenkia orientalis and Saccharomyces cerevisiae by transcriptome sequencing. *British Food Journal*, *125*(6), 1985–2001. <https://doi.org/10.1108/BFJ-06-2020-0510>
